# Supplementary material for: Intein-mediated backbone cyclization of entolimod confers enhanced radioprotective activity in mouse models
Source: PeerJ. 2018 Jun 18;6:e5043. doi: 10.7717/peerj.5043 (PMC6011820; doi:10.7717/peerj.5043)
Supplement: Supplemental Information 1 [file peerj-06-5043-s001.docx]

**Supplementary Materials**


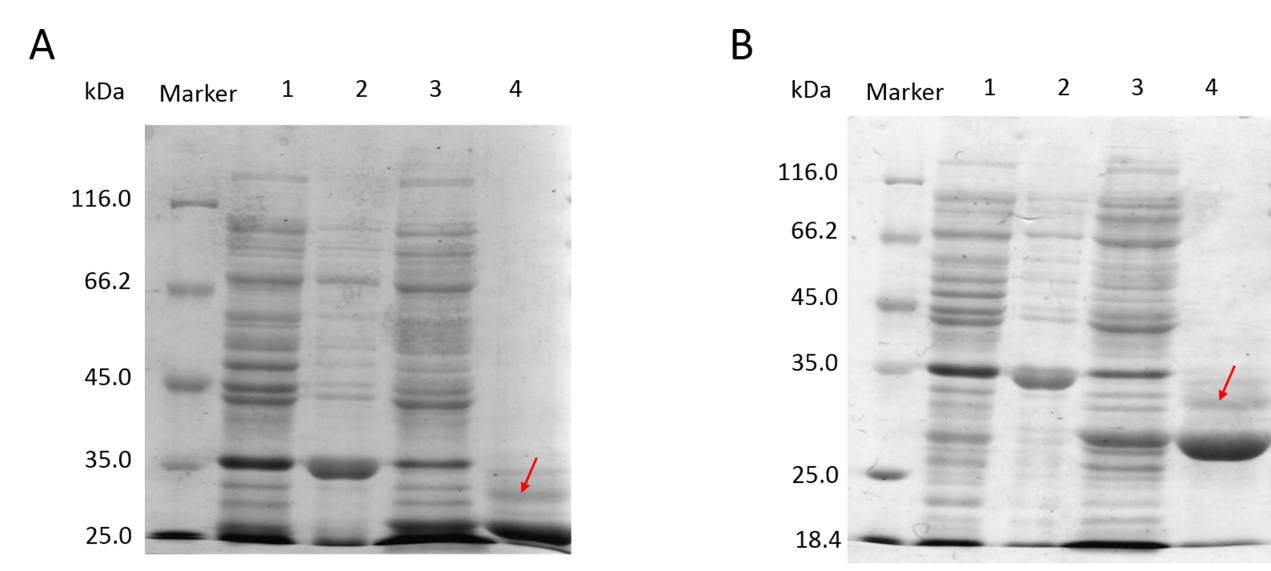


**Fig. S1. Different percentages of SDS–PAGE for separation of lin-entolimod and circ-entolimod.**

(A) 10% SDS–PAGE for separation of lin-entolimod and circ-entolimod. Lane 1: Before induction; Lane 2: lin-entolimod expression after induction with IPTG; Lane 3: Lane 3: circ-entolimod expression after induction with IPTG; Lane 4: circ-entolimod after purification. (B) 12% SDS–PAGE for separation of lin-entolimod and circ-entolimod. Lane 1: Before induction; Lane 2: lin-entolimod expression after induction with IPTG; Lane 3: Lane 3: circ-entolimod expression after induction with IPTG; Lane 4: circ-entolimod after purification.  The red arrow is not lin-entolimod.

**Q Exactive mass spectrometer (QE-MS)**

The circ-entolimod solution was adjusted to pH 8–9 with 100 mM NH_4_HCO_3_, and was then treated with 50 mM DTT, followed by 50 mM IAA. For peptides, the sample was equally divided into two parts, one for trypsin (T) digestion and the other for chymotrypsin (CT) digestion. The digested samples were diluted using solution A (2% ACN/98% H_2_O/0.1% FA), centrifuged at 20,000 × *g* at 4°C for 30 min, and the supernatant was then transferred to the Dionex (U3000) LC-QE-MS system for C-entolimod sequence analysis. The entire volume of the digested circ-entolimod solution was loaded onto a C18 trap column (C18 PepMap, 300 μm ID, 150 mm length, 300 Å pore size, 1.9 μm particle size). Mobile phase A: water with 0.1% FA, mobile phase B: 80% ACN + 0.08% FA water solution. The following elution conditions were used:

| Time(min) | 0 | 8 | 24 | 60 | 79 | 80 | 85 | 86 | 90 |
| --- | --- | --- | --- | --- | --- | --- | --- | --- | --- |
| % B | 5 | 8 | 13 | 28 | 40 | 95 | 95 | 6 | 6 |

The flow rate was 0.2 ml/min. The QE-MS parameters were as follows: data collection time, 90 min; spray voltage, 1.9 kV; capillary temperature, 320°C; normalized collision energy, 27%; molecular weight range used for collection: 300–1400 Da. Data retrieval: Mascot search engine, self-built database, first level error 15 ppm, secondary error 20 mmu.


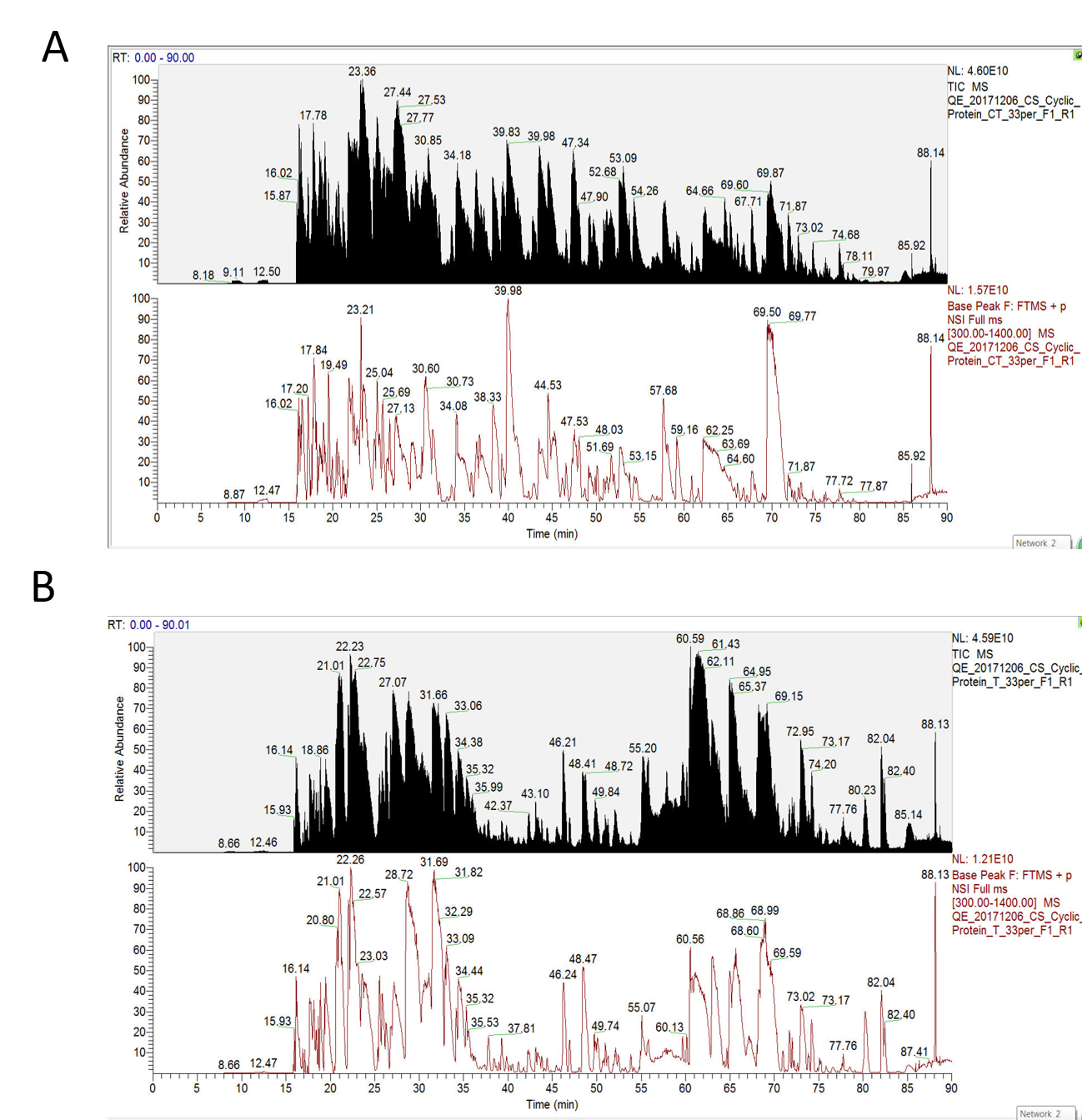


**Fig. S2. The base peak of digest.**

(A) Circ-entolimod is digested by trypsin (T). (B) Circ-entolimod is digested by chymotrypsin (CT).

The sequence coverage by T digest is

1 CFNHMWSHPQ FEKAQVINTN SLSLLTQNNL NKSQSSLSSA IERLSSGLRI

51 NSAKDDAAGQ AIANRFTSNI KGLTQASRNA NDGISIAQTT EGALNEINNN

101 LQRVRELSVQ ATNGTNSDSD LKSIQDEIQQ RLEEIDRVSN QTQFNGVKVL

151 SQDNQMKIQV GANDGETITI DLQKIDVKSL GLDGFNVNSP GISGGGGGIL

201 DSMGTLINED AAAAKKSTAN PLASIDSALS KVDAVRSSLG AIQNRFDSAI

251 TNLGNTVTNL NSARSRIEDA DYATEVSNMS KAQILQQAGT SVLAQANQVP

301 QNVLSLLRHH HHHHGS

Coverage: **98%** Score: **106137**

The sequence coverage by CT digest is

1 CFNHMWSHPQ FEKAQVINTN SLSLLTQNNL NKSQSSLSSA IERLSSGLRI

51 NSAKDDAAGQ AIANRFTSNI KGLTQASRNA NDGISIAQTT EGALNEINNN

101 LQRVRELSVQ ATNGTNSDSD LKSIQDEIQQ RLEEIDRVSN QTQFNGVKVL

151 SQDNQMKIQV GANDGETITI DLQKIDVKSL GLDGFNVNSP GISGGGGGIL

201 DSMGTLINED AAAAKKSTAN PLASIDSALS KVDAVRSSLG AIQNRFDSAI

251 TNLGNTVTNL NSARSRIEDA DYATEVSNMS KAQILQQAGT SVLAQANQVP

301 QNVLSLLRHH HHHHGS

Coverage: **91%** Score: **8248**

Combine T and CT, the coverage is 100%.


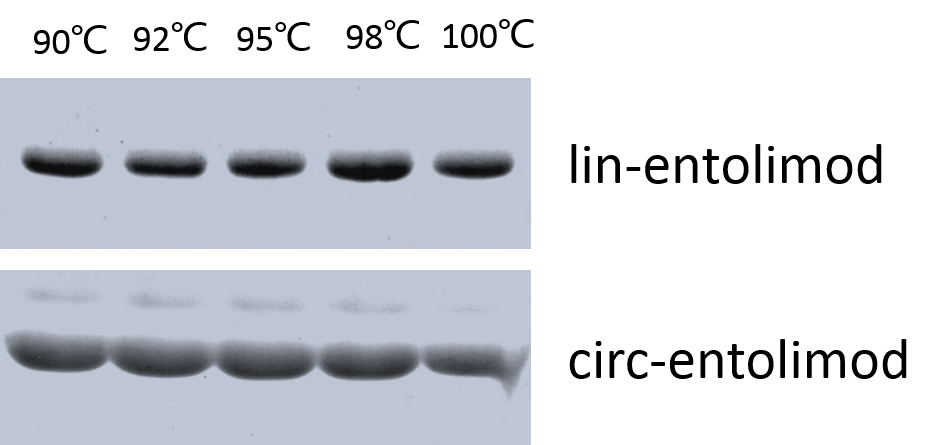


**Fig. S3. Eﬀect of heat treatment at diﬀerent temperatures on lin-entolimod and circ-entolimod.** The linear and circular form of entolimod proteins were incubated at various temperatures for 20 min (from 90 °C to 100 °C).
